# Three-dimensional heterogeneity of smooth muscle fiber density anterior to the rectum in males: quantitative analysis with implications for transanal total mesorectal excision

**DOI:** 10.1007/s00384-025-04890-1

**Published:** 2025-04-21

**Authors:** Satoru Muro, Suthasinee Tharnmanularp, Yuichiro Tsukada, Masaaki Ito, Akimoto Nimura, Keiichi Akita

**Affiliations:** 1 Department of Clinical Anatomy, Graduate School of Medical and Dental Sciences, Institute of Science Tokyo, Tokyo, Japan; 2 Princess Srisavangavadhana Faculty of Medicine, Chulabhorn Royal Academy, Bangkok, Thailand; 3 Department of Colorectal Surgery, National Cancer Center Hospital East, Chiba, Japan; 4 Department of Functional Joint Anatomy, Biomedical Engineering Laboratory, Institute of Industry Incubation, Institute of Science Tokyo, Tokyo, Japan

**Keywords:** Anal canal, Rectum, Smooth muscle, Transanal total mesorectal excision, Urethral injury

## Abstract

**Purpose:**

Transanal total mesorectal excision for rectal cancer in men poses a risk of urethral injury. The morphology of smooth muscle tissues around the rectum is reportedly characterized by differences in fiber density; however, quantitative analysis of these tissues for surgical applications is lacking. This study aimed to quantitatively analyze the histological properties of fiber density and the spatial extent of the smooth muscle anterior to the male rectum.

**Method:**

This descriptive cadaveric study involving six adult cadavers was conducted at the Tokyo Medical and Dental University. Serial histological sections were prepared from tissues in the region anterior to the rectum, and immunostaining and 3D reconstruction were performed to evaluate the spatial distribution of the smooth muscle. Smooth muscle fiber densities were measured in different regions of the smooth muscle anterior to the rectum and statistically analyzed.

**Results:**

The three-dimensional heat map revealed a gradual change in fiber density within the smooth muscle anterior to the rectum, with lower density in the superior part and higher density in the inferior part. In mid-sagittal immunostained sections, the smooth muscle anterior to the rectum exhibited a significant difference in fiber density, averaging 23.22% ± 5.50% in the superior area and significantly higher at 46.99% ± 12.92% in the inferior area.

**Conclusion:**

Heterogeneity in fiber density between the superior and inferior smooth muscle anterior to the rectum suggests that these differences could serve as landmarks, providing crucial positional information to avoid urethral injury during transanal total mesorectal excision.

**Supplementary Information:**

The online version contains supplementary material available at 10.1007/s00384-025-04890-1.

## Introduction

In recent years, the development of transanal total mesorectal excision (TaTME), a cutting-edge surgical technique for rectal cancer, has necessitated a detailed anatomical understanding of the area surrounding the rectum [1–7]. A prospective study investigating 107 surgical centers across 29 countries reported that TaTME achieves excellent short-term oncological outcomes, with R0 resection rates of 97.3% and a positive circumferential resection margin of only 2.4% [8]. In addition, the TaLaR study demonstrated that TaTME is non-inferior to laparoscopic TME (laTME) in terms of short-term surgical safety and 3-year oncological outcomes, thereby supporting its clinical relevance [9, 10]. However, the study also highlighted a significant rate of intraoperative adverse events, including a 7.8% incidence of surgeons misidentifying the correct dissection plane and a 0.7% incidence of urethral injuries. The anterior region of the male rectum lacks a clear dissection layer, making it difficult for surgeons to determine the ideal line of dissection. This presents challenges in rectal surgery because of the risk of urethral damage. We considered that a better anatomical understanding of this area would improve the precision of such surgery.

Traditionally, the anterior region of the rectum has been vaguely described as the “perineal body” [11]. However, recent studies have revealed that the region anterior to the rectum is predominantly composed of smooth muscle tissue [12–14]. This smooth muscle tissue, often referred to as the rectourethralis muscle, is continuous with the longitudinal muscle (LM) of the rectum. Its spread is not limited to the area between the rectum and the urethra but also extends laterally, sandwiching the levator ani (LA) muscle from the superior and inferior directions [15]. Additionally, studies on fetal specimens have identified the anterior rectal smooth muscle, which is innervated by the autonomic nerves of the inferior hypogastric plexus [16]. The significance of the anatomy of the smooth muscle anterior to the rectum in rectal surgery, particularly surgery using a transanal approach, has been discussed, and its visualization with ultrasound has been reported [17, 18]. However, detailed reports on the arrangement and density of the smooth muscle fibers within this smooth muscle tissue have not been made.

During TaTME in men, when dissecting the anterior side of the rectum from the anal side, the characteristics of the milky-white tissue, which is thought to be smooth muscle, change progressively from the caudal side (anal side) toward the cranial side [5]. Such intraoperative findings suggest the presence of heterogeneity within the continuous smooth muscle structure. Furthermore, our previous anatomical studies have elucidated that areas of dense and sparse smooth muscle fibers coexist within the LM layer of the anal canal, and we have reported that this unique structure can also be seen on magnetic resonance imaging [19]. We have also qualitatively demonstrated that the smooth muscle anterior to the male rectum is sparser in the superior area and denser in the inferior area [20]. These histological and anatomical findings imply that the smooth muscle around the anorectal canal is characterized by differences in density, which are reflected in imaging findings, intraoperative observations, and responses to surgical manipulations. Based on these observations, we hypothesized that there would be variations in the arrangement patterns of smooth muscle fibers depending on their location, particularly in terms of density. This aligns with previous cadaveric studies that have explored the complex anatomy of the anterior mesorectum and the Denonvilliers’ fascia, highlighting the importance of selecting the appropriate dissection plane during TME in men [21].

We considered that revealing the detailed structure of the smooth muscles surrounding the rectum will contribute to establishing the anatomical foundation necessary for improving the precision of the rectal surgery techniques for TaTME. In the current study, we sought to perform a quantitative analysis to clarify the histological properties and spatial extent of the smooth muscle anterior to the male rectum, with a focus on the density of the smooth muscle fibers.

## Methods

### Preparation of cadaveric specimens

This study was approved by the Board of Ethics at our institution (approval no. M2018-006). All methods were performed in accordance with the relevant guidelines and regulations.

Six male cadavers (mean age at death, 79.0 [range, 58–91] years) were donated to our department in accordance with the Japanese Act on Body Donation for Medical and Dental Education (Act No. 56 of 1983). All donors voluntarily declared, prior to their deaths, their intention to have their remains used as educational and study materials. Written informed consent was obtained after these donors had received a clear explanation of the purpose and methods of using donor corpses. Following their deaths, the informed consent form was explained to the bereaved families, who made no objections.

All cadavers were fixed by arterial perfusion with 8% formalin and were preserved in 30% alcohol. Cadavers with a history of pelvic organ surgery were excluded from this study.

### Standard histological analysis

Histological analysis was performed on five specimens. The pelvic region of the cadaver was sectioned in the mid-sagittal plane, and tissue blocks, measuring 20 mm × 50 mm × 5 mm, were collected from the anterior region of the rectum. After embedding in paraffin, 5-µm-thick sections were cut from the median sagittal region. Sections were stained with hematoxylin and eosin, Elastica van Gieson, and Masson’s trichrome stains. To identify smooth muscle, immunostaining was performed using an anti-smooth muscle antibody. Detailed immunostaining procedures have been described elsewhere [22].

### Wide-range serial sectioning

Wide-range serial sectioning was performed on the remaining specimen. From the area anterior to the rectum in the pelvic region of the cadaver, a large tissue block measuring 25 mm × 60 mm × 40 mm was excised. The tissue was paraffin embedded over a period approximately five times longer than usual [23]. Serial sectioning was performed at 5-µm thickness and 1-mm intervals, with 40 sections covering a width of 40 mm to the left and right, including the median sagittal plane. The sections were stained with Masson’s trichrome stain to observe the arrangement of the muscle and connective tissues. Subsequently, immunostaining using an anti-smooth muscle antibody was performed on 16 sections (each at 2-mm intervals), including the median sagittal section.

### 3D reconstruction

Using 40 sections stained with Masson’s trichrome and 16 sections immunostained for smooth muscle, obtained through wide-range serial sectioning, 3D reconstruction was performed. After aligning the positions, each structure (circular and longitudinal muscles of the anorectal canal, LA, external anal sphincter (EAS), prostate, urethra, and smooth muscle anterior to the rectum) was segmented manually, without the use of any automated algorithm. To ensure anatomical accuracy, two anatomists with expertise in the histology of the anorectal region (SM and ST) independently reviewed and confirmed all segmentation results. The segmented images were subsequently imported into TRI/3D-SRFII (ver. R.11.00.00.0-H, Ratoc, Tokyo, Japan) for 3D reconstruction [23]. The 3D reconstructed images were displayed using Mesh Lab (ver. 2022.02, ISTICNR, Rome, Italy). MeshLab is an open-access, free software platform that does not require institutional credentials and is widely accessible.

### Comprehensive analysis of 3D smooth muscle fiber density

By using 16 semi-serial sections obtained through wide-range serial sectioning at 2-mm intervals and immunostained with an anti-smooth muscle antibody, the density of the smooth muscle anterior to the rectum was comprehensively measured. Setting the *X*-axis in the left–right direction with the mid-sagittal section as *X* = 0, each section was placed at 2-mm intervals; consequently, sections on the right and left were marked as *X* =  − 2, − 4, − 6,… and *X* = 2, 4, 6,… (units in mm), respectively. The immunostained images were binarized using the ImageJ threshold function (ver. 1.53t; NIH). With the intersection of the posterior edge of the prostate and the posterior edge of the urethra in the mid-sagittal section slice set as the origin (*X* = 0, *Y* = 0, *Z* = 0), the *Y*-axis and *Z*-axis were set on each sagittal section slice. Aligning with the coordinate axes, a 2-mm square grid was placed, and the area fraction (% area) was measured at all locations where the smooth muscle was present, which was defined as the representative value of the smooth muscle fiber density within a 2-mm-diameter cubic region. From the measurement results, sagittal section heat maps, horizontal section heat maps, and 3D heat maps of fiber density were created.

### Measurement of smooth muscle density in the median sagittal section

Six mid-sagittal sections immunostained for smooth muscle (five from standard histological analysis and one from wide-range serial sectioning) were used to measure the fiber density of the smooth muscle anterior to the rectum. The immunostained images were binarized using the ImageJ threshold function (ver. 1.53t; NIH, Bethesda, MD, USA). The region of smooth muscle anterior to the rectum was divided into three areas—superior, middle, and inferior—by dividing the vertical span into thirds. In each of these areas, the area fraction (% area) was measured and defined as the density of smooth muscle fibers.

### Statistical analysis

The collected fiber density data were subjected to analysis of variance (ANOVA) to assess statistically significant differences across the three measurement areas: superior, middle, and inferior. This analysis was performed using Python (version 3.11.8) with the scipy library for ANOVA and the statsmodels library for Tukey’s honestly significant difference (HSD) test, with the significance threshold set at *p* < 0.05. After observing significant differences from the ANOVA, Tukey’s HSD test was employed for post hoc analysis to pinpoint specific area comparisons that exhibited significant variance in fiber density.

## Results

**Fig. 1:**
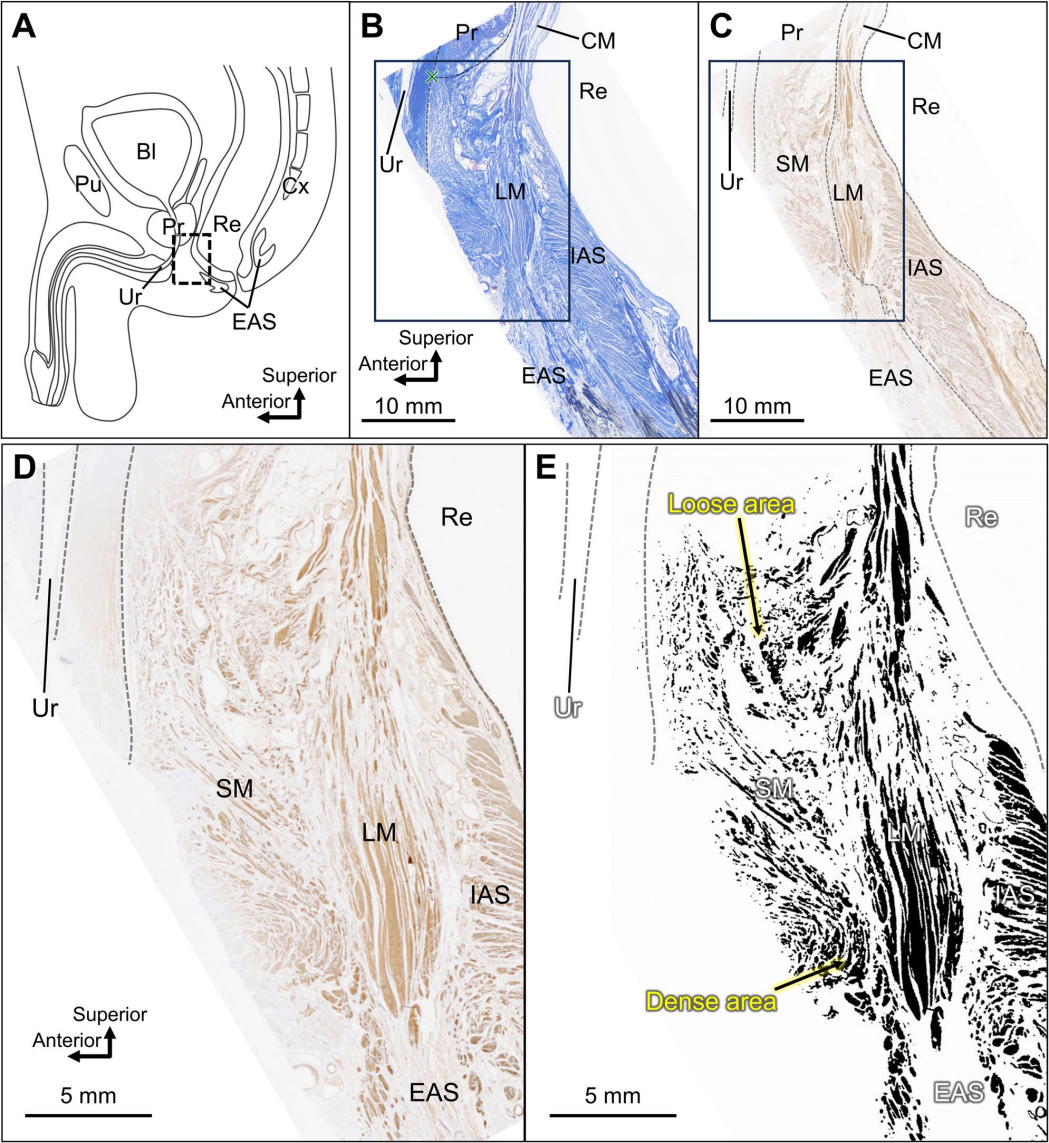
Histological analysis of the mid-sagittal section of the region anterior to the rectum. **A** Schematic of the mid-sagittal section of the male pelvis, with the region anterior to the rectum from which histological samples were obtained as indicated by a dotted square. **B** Masson’s trichrome staining was performed. The muscles of the anorectal canal (IAS, LM, and EAS) are observable. The urethra is located approximately 10 mm anterior to the border of the rectal wall. **C** Immunostaining of smooth muscles confirms that the tissue between the rectum and urethra is primarily composed of smooth muscles. **D** Enlarged view of the rectangular frame as shown in **C**. **E** Binarized version of the image as shown in **D**. The smooth muscle fibers appeared loose in the superior area and dense in the inferior area. Bl, bladder; CM, circular muscle; Cx, coccyx; EAS, external anal sphincter; IAS, internal anal sphincter; LM, longitudinal muscle; Pr, prostate; Pu, pubis; Re, rectum; SM, smooth muscle anterior to the rectum; Ur, urethra

In the mid-sagittal section, the region anterior to the rectum was observed histologically (Fig. 1). The circular muscle (IAS), LM, and EAS were observed from the luminal side of the anorectal canal (Fig. 1B). The urethra was located approximately 10 mm anterior to the anterior edge of the LM, that is, the anterior border of the rectal wall. The tissue between the rectum and the urethra was immunohistologically shown to be smooth muscle, and this smooth muscle tissue was widely spread in the anterior region of the rectum (Fig. 1C). The smooth muscle filled the space where the rectum and urethra were closest to each other, and it inferiorly extended anteriorly to the EAS. Under high magnification and in binarized images, heterogeneity in the density within the smooth muscle anterior to the rectum was observed (Fig. 1D and E). Qualitative assessment showed that the smooth muscle fibers appeared loose in the superior area and dense in the inferior area.

**Fig. 2:**
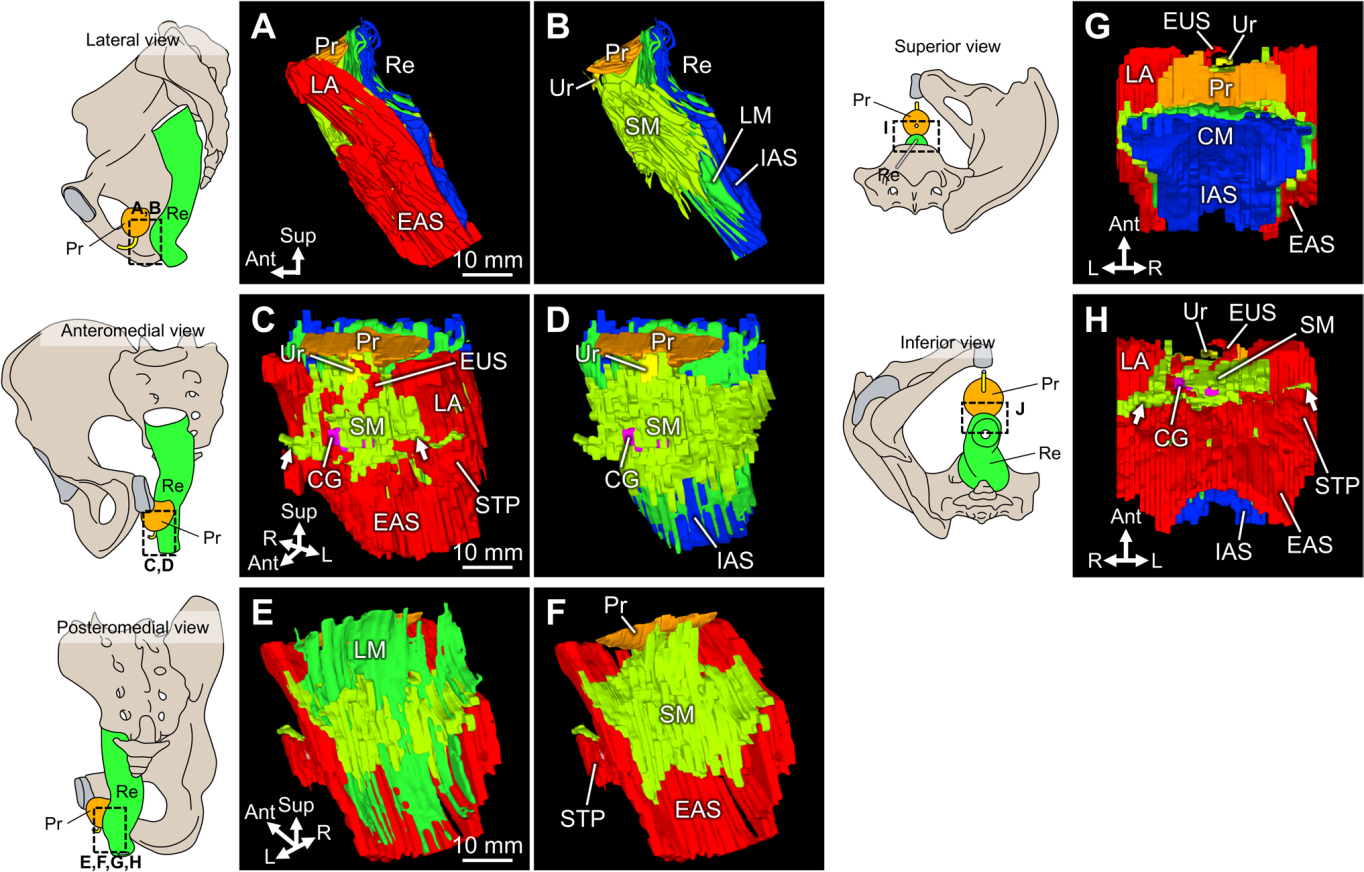
3D reconstruction of the smooth muscle anterior to the rectum. Schemas of the rectum and prostate from various orientations are placed on the left side of each figure. The dotted rectangle in the schemas corresponds to the orientation of the 3D images as shown in **A**–**H**. **A** Lateral aspect. The LA descends obliquely along the prostate and joins the EAS on the rectal side. **B** After removal of the skeletal muscles (LA and EAS), the smooth muscle tissues (SM) are seen to expand anteriorly to the rectum. **C** Aspects viewed anteriorly. The SM is located behind Cowper’s glands, spreading bilaterally anteroinferior to the LA (indicated by arrows). **D** After removing the skeletal muscles (LA and EAS), the SM is localized immediately behind the urethra. **E** View from the posterior (luminal rectal) side. The IAS is already removed, and the LM can be observed. **F** Upon removal of the LM, the smooth muscle anterior to the rectum (SM) is visible. The SM spreads on the superior inner surface of the LA. **G** View from the superior side. The urethra is located approximately 10 mm anterior to the rectal wall. **H** View from the inferior side. The space between the rectum and urethra is occupied by the SM. CG, Cowper’s gland; CM, circular muscle; EAS, external anal sphincter; EUS, external urethral sphincter; IAS, internal anal sphincter; LA, levator ani; LM, longitudinal muscle; Pr, prostate; Re, rectum; SM, smooth muscle anterior to the rectum; STP, superficial transverse perineal muscle; Ur, urethra

The 3D smooth muscle anterior to the rectum was visualized to extend between the bilateral LA muscles, rectum, and urethra and was inferior to these structures (Fig. 2, Supplementary 3D PDF). When viewed anteroinferiorly, the smooth muscle anterior to the rectum was located behind Cowper’s gland and spread laterally anteroinferior to the LA (arrows in Fig. 2C and H). Viewed posteriorly, the smooth muscle was located anterior to the LM, spreading between the rectum and urethra, further posteriorly, and above the LA muscles (Fig. 2E and F).

**Fig. 3:**
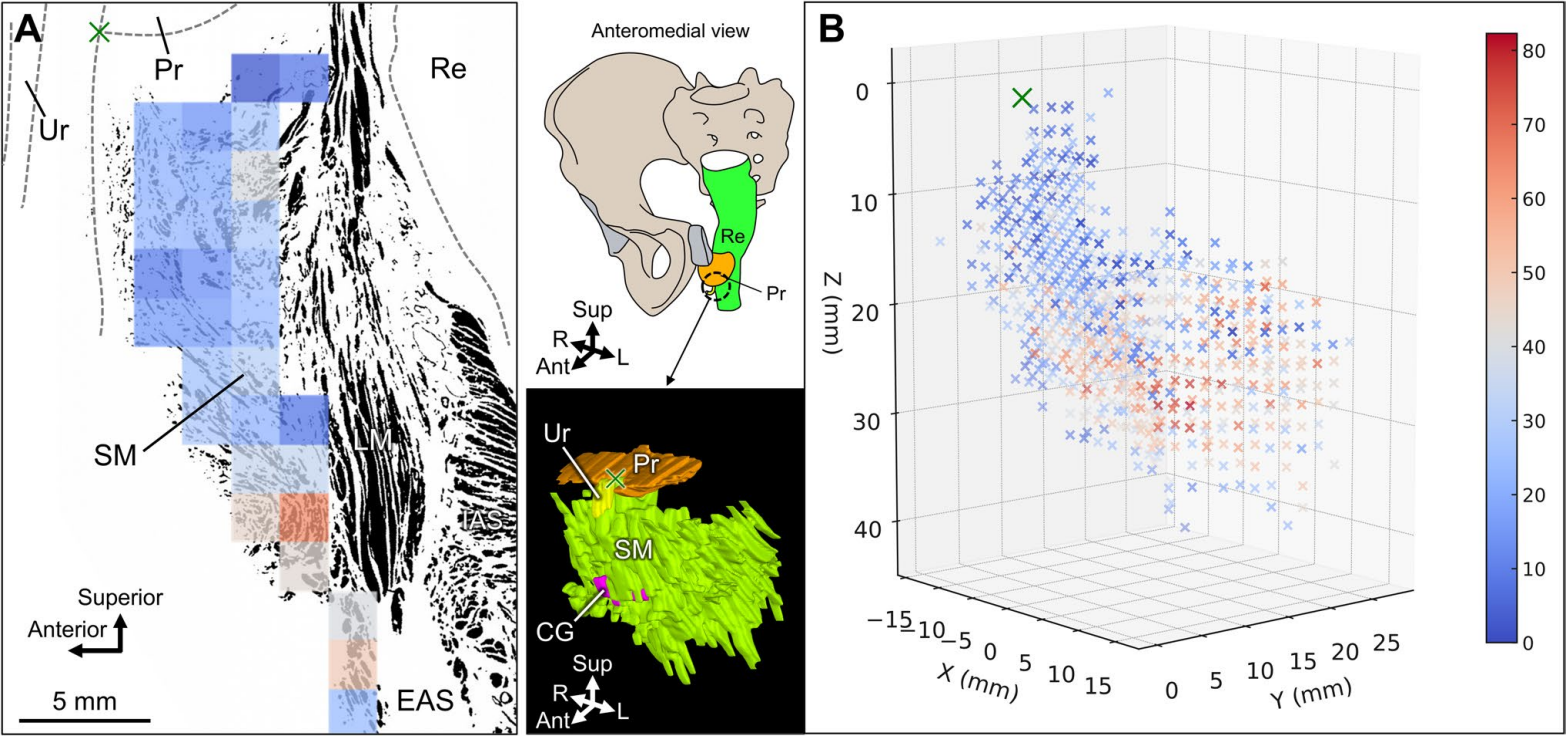
Sagittal and 3D heat maps of smooth muscle fiber density. **A** The binarized image of smooth muscle fibers in a mid-sagittal section, overlaid with a density heat map, in which red indicates high density and blue indicates low density. The smooth muscle anterior to the rectum has lower density (looser) in the superior region and higher density (denser) in the inferior region. The intersection of the posterior edge of the prostate and the posterior edge of the urethra in the mid-sagittal section slice is set as the origin (*X* = 0, *Y* = 0, and *Z* = 0) (indicated by the green cross). **B** Three-dimensional heat map constructed from the measurement results of smooth muscle fiber density in semi-serial sections. The schemas and 3D images on the left indicate the angles and ranges represented by the 3D heat map. Within the three-dimensionally spreading smooth muscle anterior to the rectum, the superior part demonstrated low-density (loose) fibers, and the inferior part demonstrated high-density (dense) fibers. EAS, external anal sphincter; IAS, internal anal sphincter; LM, longitudinal muscle; Pr, prostate; Re, rectum; SM, smooth muscle anterior to the rectum; Ur, urethra

Figure 3A shows a binarized image of smooth muscle fibers in a mid-sagittal section overlaid with a density heat map. Comprehensive quantitative analysis also demonstrated that the smooth muscle anterior to the rectum had lower density (looser) in the superior region and higher density (denser) in the inferior region (Fig. 3A). The 3D heat maps also demonstrated that within the 3D spreading smooth muscle tissue anterior to the rectum, the superior tissue had a low density, while the inferior tissue had a high density of muscle fibers (Fig. 3B). The heat maps of the horizontal sections also showed that the density of the smooth muscle fibers was lower in the superior part and higher in the inferior part (Online Resource 1). In the superior part, an area without smooth muscle fibers was seen between the smooth muscle anterior to the rectum and the LA (asterisks in Online Resource 1).

**Fig. 4:**
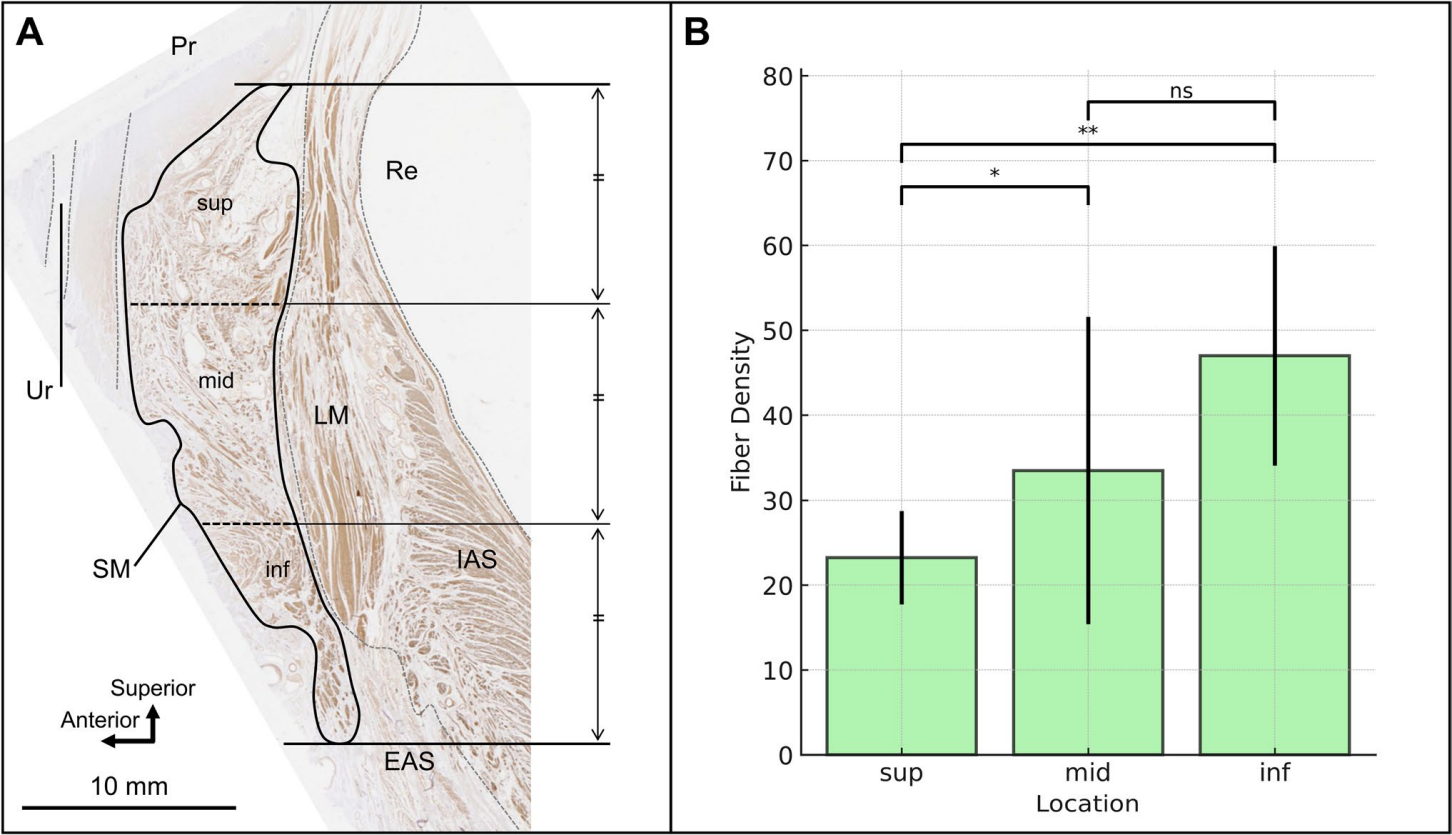
Fiber density across different areas in the smooth muscle anterior to the rectum. **A** Histological mid-sagittal section immunostained for smooth muscle fibers. The smooth muscle anterior to the rectum was divided into three areas—superior (sup), middle (mid), and inferior (inf)—by dividing the vertical span into thirds. In each of these areas, the area fraction (% area) was measured and defined as the density of smooth muscle fibers. **B** The bar chart represents the mean fiber density (± standard deviation) for the three measurement areas: superior, middle, and inferior. Significant differences were observed between the inferior and middle regions (*p* = 0.041), as well as between the inferior and superior regions (*p* = 0.002). Significant differences are indicated by asterisks: *p < 0.05, **p < 0.01. No significant difference is indicated by “ns.” EAS, external anal sphincter; IAS, internal anal sphincter; LM, longitudinal muscle; Re, rectum; SM, smooth muscle anterior to the rectum; Ur, urethra

In the mid-sagittal immunostained sections, the average density of the smooth muscle fiber was 23.22% ± 5.50% in the superior region, 33.49% ± 18.09% in the middle region, and 46.99% ± 12.92% in the inferior region (Fig. 4), showing significance difference (F(2, 15) = 4.88, *p* = 0.023). Subsequent Tukey’s HSD post hoc analysis revealed significant differences between the inferior and middle regions (*p* = 0.041) and between the inferior and superior regions (*p* = 0.002). However, no significant difference was observed between the middle and superior regions (*p* = 0.193). This analysis indicates that the muscle fiber density in the inferior region is significantly higher than that for both the middle and superior regions.

## Discussion

### Summary of principal findings

In this study, based on histological data, the 3D spatial extent of the smooth muscle anterior to the rectum was visualized, and the density of smooth muscle fibers was comprehensively analyzed quantitatively. The smooth muscle spreads between the rectum and urethra, above the LA, and laterally anteroinferior to the LA. The measurement results underscored the heterogeneity in smooth muscle fiber density within the smooth muscle structures surrounding the rectum. The fiber density was significantly lower in the superior area than in the inferior area of the smooth muscle anterior to the rectum.

### The fiber density of the smooth muscle anterior to the rectum

The novelty of this study lies in its quantitative description of the histological characteristics of the smooth muscle anterior to the rectum. The region anterior to the rectum, conventionally vaguely described as the “perineal body” [9, 24, 25], has recently been reported to be occupied by smooth muscle tissue [12–14]. This area, mainly referred to as the rectourethralis muscle, is known for its spatial expansion, continuity with the LM, attachment to the posterior wall of the urethra, and lateral spreading to enclose the LA superiorly and inferiorly [15, 17, 18, 26]. However, the histological characteristics of the smooth muscle anterior to the rectum have not been fully described to date, and surgical landmarks are scarce. This study quantified the fiber density of the smooth muscle anterior to the rectum, which had not been reported previously, and statistically demonstrated the differences in density between the superior and inferior parts.

### Utility of recognizing smooth muscle fiber density differences during TaTME

Although TaTME has shown promising outcomes in recent studies, including the TaLaR trial [9, 10], concerns remain regarding intraoperative complications, such as urethral injury. In this context, the results of this study suggest that differences in smooth muscle density can serve as landmarks for intraoperative craniocaudal positioning.

**Fig. 5:**
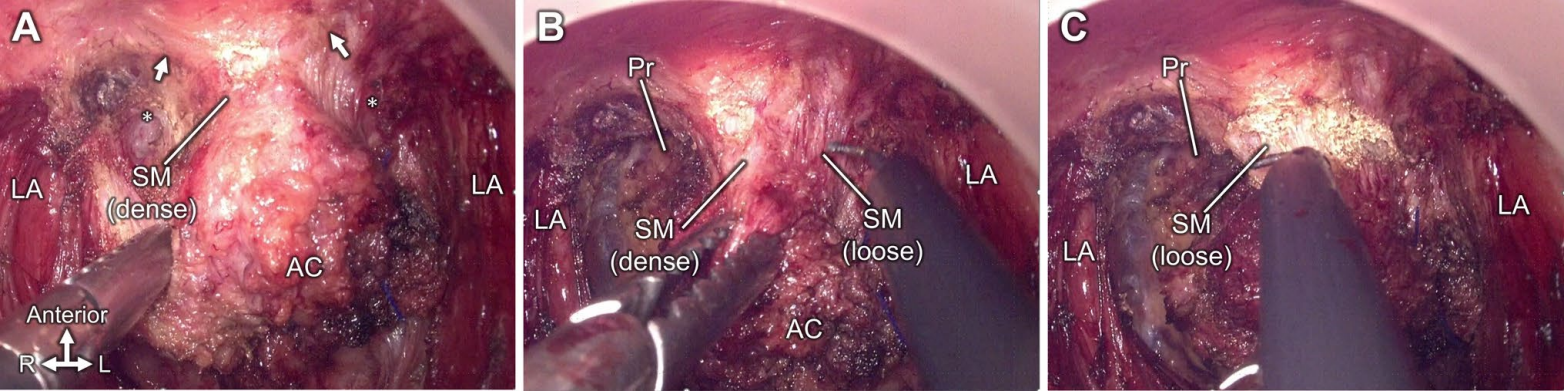
Intraoperative observation showing the dissection of the anterior wall of the rectum during transanal total mesorectal excision. **A** Dense milky-white tissue observed when cutting through the anterior rectal wall from the perineal side is identified as the inferior dense area of SM. The SM spread laterally anteroinferior to the LA (indicated by arrows). Superolateral to the SM is an easily separable space lacking smooth muscle tissue (indicated by asterisks). **B** While cutting through the SM, low-density loose tissue is revealed, which is identified as the superior loose area of SM. **C** Further cutting through the SM reveals a low-density superior area characterized by sparse fibers that become directional upon traction. AC, anal canal; Pr, prostate; LA, levator ani; SM, smooth muscle anterior to the rectum

The results of this study suggest that differences in smooth muscle density can serve as landmarks for intraoperative craniocaudal positional information. In TaTME in males, cutting the anterior rectal wall is challenging because of the lack of a clear dissection layer and the risk of urethral injury. During actual TaTME surgery, when cutting through the anterior rectal wall from the perineal side, dense, milky-white tissue is observed (Fig. 5A), which, in light of this study, is considered the inferior area of the smooth muscle. This finding is consistent with the observation that this tissue spreads laterally and anteroinferiorly to the LA (arrows in Fig. 5A). Cutting through this dense tissue gradually revealed looser, less dense tissue (Fig. 5B and C), which is the superior area of the smooth muscle. Thus, the difference in smooth muscle density can help differentiate between the inferior and superior areas of the smooth muscle that have been cut. Additionally, because the urethra is located immediately anterior to the superior loose area of smooth muscle, its proximity to the urethra serves as a warning landmark. Surgeons need to observe the density of the smooth muscle. When looser and less dense smooth muscle tissue appears during surgery, they should be aware that the urethra is immediately anterior to the dissection plane when deciding on the direction of dissection. On the other hand, the risk of urethral injury is low as long as they are cutting denser smooth muscle tissue. However, because the smooth muscle anterior to the rectum is a continuous structure with the rectal wall and is difficult to distinguish, it is necessary to be cautious to minimize the risk of rectal wall injury by getting too close to the rectal wall. Such anatomical landmarks may help to reduce the misidentification of the correct dissection plane and the incidence of urethral injuries in TaTME. Moreover, since an area without smooth muscle tissue is present between the smooth muscle and the LA, starting the dissection from this smooth muscle-free area near the midline, before approaching the central smooth muscle tissue, can help determine the dissection layer (Fig. 5). Such a loose area that is sparse in smooth muscle fibers and is located paracentrally has also been reported in women [27].

Currently, smooth muscle density cannot be measured preoperatively in clinical settings. However, future advancements in MRI may allow for such an assessment, since we have previously reported that smooth muscle density in the anal canal is correlated with MRI signal intensity [19]. During TaTME, surgeons can qualitatively evaluate smooth muscle density endoscopically. The relative change—from dense to sparse muscle—could serve as a useful intraoperative marker to judge dissection depth and proximity to the urethra, thus helping to avoid injury. These findings may also inform surgical planning beyond TaTME. In cases with locally advanced tumors in the superior third of the LM layer, the anatomical complexity may hinder distal dissection, regardless of the approach adopted. Therefore, recognizing such positional variations may help surgeons anticipate technical difficulties in standard TME and guide the selection of the most appropriate surgical strategy.

### Significance of the 3D visualization of smooth muscle tissue and the quantification of fiber density

The strength of this study lies in the immunohistochemical detection of smooth muscle fibers coupled with 3D visualization and quantitative analysis. The area between the rectum and urethra is complexly arranged with intertwined skeletal muscles of the pelvic floor and smooth muscles, making the anatomical structure difficult to grasp. By performing 3D reconstruction, the 3D extent of the tissue was clarified. Additionally, by using the fiber density parameter, which influences muscle function, intraoperative findings, and dissectability, this study revealed anatomically and clinically significant tissue characteristics.

One limitation of this study is that it involved an analysis of cadavers exclusively and did not include living subjects. Future studies should aim to analyze living bodies in light of this research by using intraoperative videos, computed tomography, and magnetic resonance imaging. In addition, the integration of artificial intelligence (AI) with 3D anatomical reconstructions represents a promising avenue for future development. Machine learning models trained on real surgical cases could assist in recognizing anatomical patterns and enhance intraoperative navigation, especially when combined with augmented reality (AR) technologies. Moreover, the combination of preoperative imaging omics with 3D anatomical reconstructions may serve as a powerful tool for personalized surgical planning. By integrating the anatomical insights gained from this study, such technologies could help surgeons to better anticipate intraoperative challenges and tailor their approach to individual patients. These precision-oriented strategies have the potential to improve not only intraoperative safety but also postoperative outcomes.

### Unanswered questions and future research

Fibrous tissues can be characterized by their density and the regularity of orientation [28]. This study successfully quantified the fiber density differences in the smooth muscle anterior to the rectum, providing quantitative support for previously reported qualitative findings [20]. By doing so, this study reinforces the earlier observations with new quantitative data. Understanding the differences in fiber orientation is also crucial for improving decision-making in endoscopic surgery. Therefore, future research should focus on quantitatively analyzing these orientation differences to further enhance surgical outcomes.

## Conclusions

The 3D extent of the smooth muscle anterior to the rectum and the heterogeneity in smooth muscle fiber density demonstrated in this study could explain the intraoperative findings during TaTME. The varying fiber densities between the superior and inferior areas of this smooth muscle suggest that these differences could serve as landmarks for craniocaudal positional information during surgery. Furthermore, the significantly lower fiber density in the superior area of this smooth muscle compared to that in the inferior area suggests that changes in fiber density during anterior rectal wall dissection could serve as an indicator to avoid urethral damage during TaTME.

## Supplementary Information

Below is the link to the electronic supplementary material.

Supplementary file1 Mid-sagittal and horizontal heat maps of smooth muscle fiber density, A: Heat map of smooth muscle fiber density in the mid-sagittal section of the smooth muscle anterior to the rectum. Red indicates high density (dense) and blue indicates low density (loose). Dotted lines correspond to the horizontal sections B–G. B: Horizontal section of the superior smooth muscle anterior to the rectum. The smooth muscle between the rectum and urethra has low fiber density (loose). Some areas in the paramedian lateral region lack smooth muscle (asterisks). C: 4 mm inferior to B. D: 4 mm inferior to C. In the area between the rectum and bilateral LAs, the fiber density of the smooth muscle is higher than that in the superior sections. E: 4 mm inferior to D. F: 4 mm inferior to E. The smooth muscle fiber density of the smooth muscle anterior to the rectum is higher than that in the superior sections. G: 4 mm inferior to F. CM, circular muscle; EAS, external anal sphincter; EUS, external urethral sphincter; IAS, internal anal sphincter; LA, levator ani; LM, longitudinal muscle; Pr, prostate; Re, rectum; STP, superficial transverse perineal muscle; Ur, urethra (TIF 11666 KB)

ESM 2 (PNG 2.73 MB)

Supplementary file2 Three-dimensional-reconstructed image based on serial histological sections. It was designed for interactive exploration in Adobe Acrobat Reader (https://www.adobe.com/), enabling users to rotate, zoom, and slice through the visualization. (PDF 1447 KB)

## Data Availability

No datasets were generated or analysed during the current study.
