# Supplementary figures and images for: Three-dimensional heterogeneity of smooth muscle fiber density anterior to the rectum in males: quantitative analysis with implications for transanal total mesorectal excision
Source: Int J Colorectal Dis. 2025 Apr 21;40(1):95. doi: 10.1007/s00384-025-04890-1 (PMC12011932; doi:10.1007/s00384-025-04890-1)

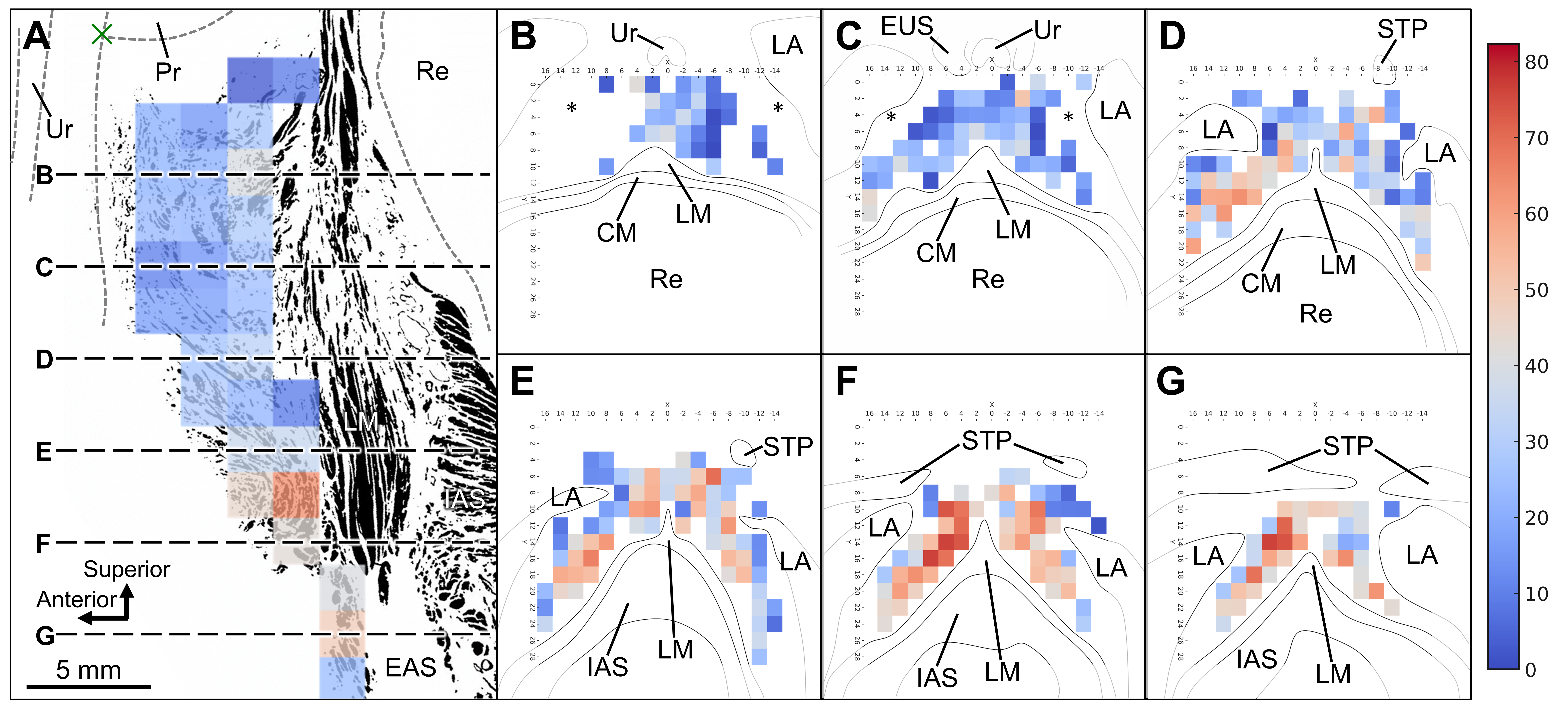

Supplement: Supplementary file 1 — Supplementary file1 Mid-sagittal and horizontal heat maps of smooth muscle fiber density, A: Heat map of smooth muscle fiber density in the mid-sagittal section of the smooth muscle anterior to the rectum. Red indicates high density (dense) and blue indicates low density (loose). Dotted lines correspond to the horizontal sections B–G. B: Horizontal section of the superior smooth muscle anterior to the rectum. The smooth muscle between the rectum and urethra has low fiber density (loose). Some areas in the paramedian lateral region lack smooth muscle (asterisks). C: 4 mm inferior to B. D: 4 mm inferior to C. In the area between the rectum and bilateral LAs, the fiber density of the smooth muscle is higher than that in the superior sections. E: 4 mm inferior to D. F: 4 mm inferior to E. The smooth muscle fiber density of the smooth muscle anterior to the rectum is higher than that in the superior sections. G: 4 mm inferior to F. CM, circular muscle; EAS, external anal sphincter; EUS, external urethral sphincter; IAS, internal anal sphincter; LA, levator ani; LM, longitudinal muscle; Pr, prostate; Re, rectum; STP, superficial transverse perineal muscle; Ur, urethra (TIF 11666 KB) [file 384_2025_4890_MOESM1_ESM.tif]

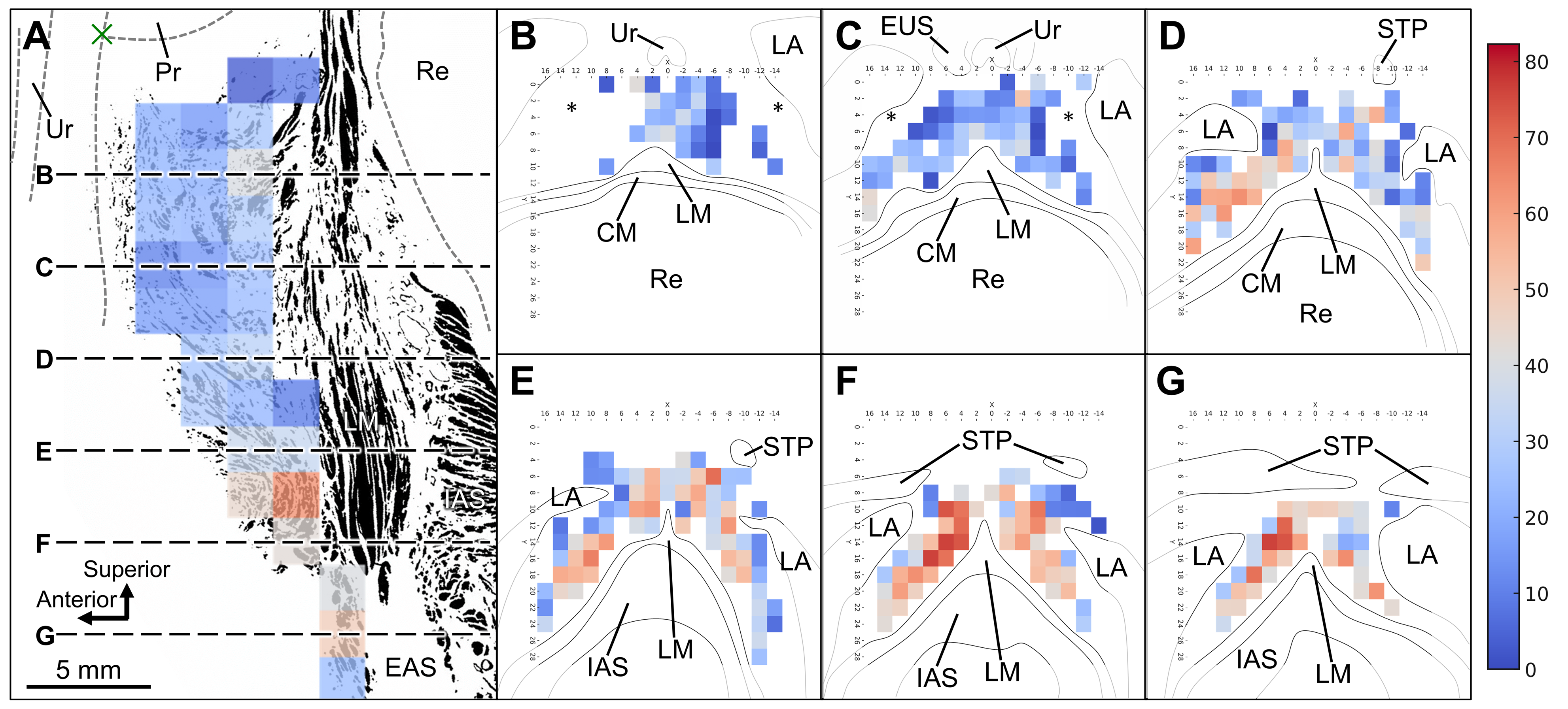

Supplement: Supplementary file 2 — (PNG 2.73 MB) [file 384_2025_4890_Fig6_ESM.png]
